# Can Life Storybook-based Group Reminiscence Therapy Benefit Older Adults With Mild Cognitive Impairment?

**DOI:** 10.1093/geroni/igaf122.3402

**Published:** 2025-12-31

**Authors:** Minhee Yang, Eunhee Cho, Kyung Hee Lee, Bada Kang, Chang Gi Park, Jun-Ah Song

**Affiliations:** Yonsei University, Seoul, Republic of Korea; Yonsei University, Seoul, Republic of Korea; Yonsei University, Seoul, Republic of Korea; Yonsei University College of Nursing, Seoul, Republic of Korea; University of Illinois Chicago, Chicago, Illinois, United States; Korea University, Seoul, Republic of Korea

## Abstract

Preventing dementia in older adults with mild cognitive impairment (MCI) is critical; however, existing interventions often overlook psychosocial well-being. Life storybook (LSB)-based group reminiscence therapy (RT) provides a structured and engaging approach to support both cognitive and psychosocial health. This cluster-randomized trial aimed to evaluate the effectiveness of LSB-based group RT on cognitive function, self-esteem, depression, and social interaction among community-dwelling older adults with MCI in South Korea. Thirty-six participants were randomly assigned to either a 6-week LSB-based group RT or a control group with self-directed dementia prevention training materials. Data from 31 participants, after accounting for dropouts, were analyzed using a linear mixed-effects model. The intervention group showed significantly greater self-esteem and cognitive function improvements than the control group. However, no significant changes were observed in depression or social interaction. Qualitative findings suggested that the LSB-based group RT fostered engagement, empathy, and mutual support, potentially enhancing social interaction within the group beyond the scope of quantitative measures. While the small sample size of this pilot study may limit generalizability, these findings highlight the potential of the LSB-based group RT as an effective community-based intervention for promoting cognitive and psychosocial well-being in older adults with MCI. Future research should explore its long-term effects and broader implementation to inform innovative dementia prevention strategies.

